# 512. Interim Results of a Prospective Cohort Study to Monitor the Emergence of Resistance in Immunocompromised Non-Hospitalized Patients With COVID-19 Who Were Treated with Sotrovimab in Great Britain: LUNAR Study 

**DOI:** 10.1093/ofid/ofad500.581

**Published:** 2023-11-27

**Authors:** Judith Breuer, Myriam Drysdale, Jill Walker, Jennifer Han, Magdalena Gorczycka, Alicia Aylott, Melissa K Van Dyke, Helen Birch, Elizabeth McKie, David M Lowe

**Affiliations:** UCL, London, United Kingdom; GSK, Brentford, Middlesex, England, United Kingdom; GlaxoSmithKline, Cambridge, Massachusetts; GlaxoSmithKline, Cambridge, Massachusetts; GSK, Brentford, Middlesex, England, United Kingdom; GSK, Brentford, Middlesex, England, United Kingdom; GSK, Brentford, Middlesex, England, United Kingdom; GSK, Brentford, Middlesex, England, United Kingdom; GSK, Brentford, Middlesex, England, United Kingdom; UCL, London, United Kingdom

## Abstract

**Background:**

Immunocompromised (IC) patients are at risk of adverse COVID-19 outcomes. The risk of treatment emergent resistance may be high in this population. This study investigated clinical and virological outcomes in sotrovimab-treated IC patients in Great Britain while the Omicron variant was predominant.

**Methods:**

IC, non-hospitalized patients aged ≥ 18 years who were infected with SARS-CoV-2 and received early treatment with sotrovimab 500 mg IV for COVID-19 as per standard of care were included in this multicenter, single arm, observational prospective cohort study. Nasal/oropharyngeal samples were collected at baseline, Day (D) 7, 14, and 28 (+/-2 days) for viral load and sequencing analyses. Clinical (hospitalization, respiratory support, intensive care unit [ICU] admission and death) and safety outcomes were assessed through D28. This interim analysis included patients enrolled from 1 July 2022–31 January 2023.

**Results:**

Among 195 patients (median age: 58 years), 56% were female, 86% were white, and 98.5% had ≥ 1 COVID-19 vaccine dose prior to enrollment. All patients received sotrovimab within 8 (median: 2) days of diagnosis. Absolute median viral load declined from 7.46 log_10_ copies/mL at baseline to 0.55 log_10_ copies/mL at D28 (**Table 1**). Of 189 patients with spike consensus sequencing data, all harbored the Omicron variant, with 32 sublineages identified. Omicron BA.4, BQ.1, BE.9, BA.5.1.18, and BN.1 were most common in this data cut. We also plan to present interim data on treatment emergent substitutions. No patients were hospitalized due to COVID-19. Six (1.3%) patients had all-cause hospitalizations; none were admitted to ICU. One patient (0.5%; infected with Omicron CH.1.1) with progressive neuromuscular disease needed high flow oxygen/non-invasive mechanical intervention and died on D18 (death deemed not COVID-19 related by investigator) (**Table 2**). Three mild sotrovimab-related adverse events were reported.

**Table 1:**
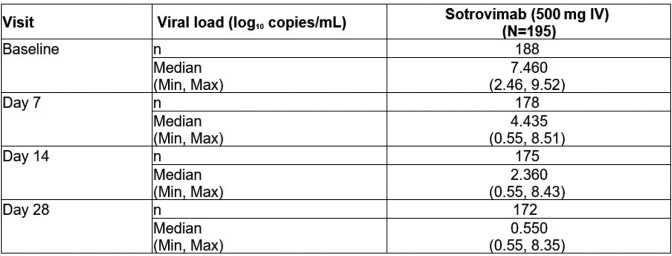
Summary of absolute viral load (log10 copies/mL) through Day 28, as measured by qRT-PCR from nasal/oropharyngeal swabs Note: Baseline log10 viral load was defined as the non-missing assessment taken at Day 0, excluding the negative viral load results. The post-baseline viral load values with Ct value > 38 were imputed as half the lower limit of detection (i.e. 453 copies/mL*0.5=226.5). Negative viral loads were defined as Ct ≥ 45 and were imputed as 3.57 copies/mL. These imputed values were used to derive log10 viral loads. Ct, cycle threshold; IV, intravenous; qRT-PCR, quantitative real-time reverse transcriptase polymerase chain reaction

**Table 2:**
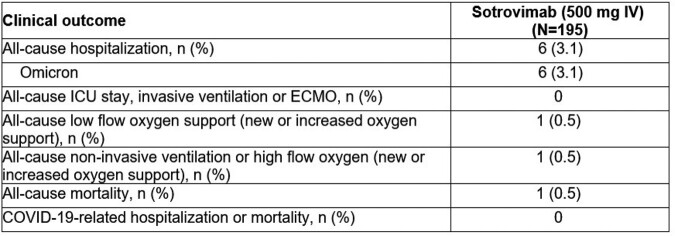
Summary of clinical outcomes through Day 28 Notes: N = Number of participants in Completer population (defined as all participants who were enrolled, exposed to study intervention [sotrovimab], and who had been followed for 30 days or discontinued from the study early); n = Number of participants with data available. ECMO, extracorporeal membrane oxygenation; ICU, intensive care unit; IV, intravenous

**Conclusion:**

Sotrovimab-treated patients had reduced viral load by D7 which further decreased through D28, despite being IC and infected with Omicron subvariants (reduced in vitro neutralization has been reported for some of the subvariants). Few severe clinical outcomes were reported (all unrelated to COVID-19).

**Disclosures:**

**Judith Breuer, MD**, GSK: Funding to conduct study|MRC/UKRI: Grant/Research Support|National Institutes of Health: Grant/Research Support|NIHR: Senior investigator|UKNIHR: Grant/Research Support|Vir Biotechnology, Inc: Funding to conduct study|Wellcome Trust: Grant/Research Support **Myriam Drysdale, PhD**, GSK: Employee|GSK: Stocks/Bonds **Jill Walker, PhD**, GSK: Employee|GSK: Stocks/Bonds **Jennifer Han, MD**, GSK: Employment|GSK: Stocks/Bonds **Magdalena Gorczycka, MSc**, GSK: Employee|GSK: Stocks/Bonds **Alicia Aylott, MSc**, GSK: Employee|GSK: Stocks/Bonds **Melissa K. Van Dyke, PhD**, GSK: Employee|GSK: Stocks/Bonds **Helen Birch, PhD**, GSK: Employee|GSK: Stocks/Bonds **Elizabeth McKie, PhD**, GSK: Employee|GSK: Stocks/Bonds **David M. Lowe, FRCP, PhD**, Biotest: Speaker fees|Blood Cancer UK: Grant/Research Support|Bristol Myers Squibb: Grant/Research Support|British Society of Antimicrobial Chemotherapy: Grant/Research Support|Gilead: Speaker fees (educational video)|GSK: Funding to conduct study|Langland: Speaker fees|LifeArc: Grant/Research Support|Merck: Personal fees for a round-table discussion|National Institute of Health Research: Grant/Research Support|Octapharma: Travel, accommodation and conference fees|UK Medical Research Council: Grant/Research Support|Vir Biotechnology, Inc: Funding to conduct study

